# Assessing pain management in total joint arthroplasty using the Detroit interventional pain assessment scale—A prospective cohort study

**DOI:** 10.1186/s42836-024-00276-w

**Published:** 2024-11-01

**Authors:** Lauryn J. Boggs, Ishan Patel, Melina Holyszko, Bryan E. Little, Hussein F. Darwiche, Rahul Vaidya

**Affiliations:** 1 Department of Orthopedic Surgery, Wayne State University School of Medicine, 540 E. Canfield Ave., Detroit, MI 48201 USA; 2 Department of Orthopaedics, Detroit Medical Center, 311 Mack Ave 5Th Floor, Detroit, MI 48201 USA

**Keywords:** Total Joint Arthroplasty, Pain Assessment, Detroit Interventional Pain Assessment Scale, Opioids, Morphine Milligram Equivalents

## Abstract

**Background:**

Total joint arthroplasty (TJA) is an effective treatment for end-stage osteoarthritis, but postoperative pain has been poorly managed. The purpose of this study was to (1) assess how much narcotic medication was prescribed after TJA; (2) assess if patients were satisfied with their pain management; (3) compare these same data between total hip arthroplasty (THA)/total knee arthroplasty (TKA); (4) compare these same data between preoperative opioid users/opioid-naïve patients.

**Methods:**

An IRB-approved prospective study was conducted at a US academic joint replacement practice. Patients were evaluated by an independent observer at three weeks, three months, and six months postoperatively using the Detroit Interventional Pain Assessment (DIPA) scale. Patients verbally rated their pain with their current medication regimen as 0 (no pain), 1 (tolerable pain), or 2 (intolerable pain) on the DIPA scale. Narcotic usage was verified by the Michigan Automated Prescription System (MAPS). Patients were divided into THA, TKA, previously on opioids, and opioid-naïve groups. Provider efficiency scores reflected pain management satisfaction and were calculated as the percentage of patients reporting no pain or tolerable pain.

**Results:**

Out of 200 patients, the percentage of patients using narcotics and their daily usage (MMEs) significantly decreased from 75.5% (27.5 MMEs) at three weeks to 42.9% (5.3 MMEs) at six months (*P* < 0.001). In 80% of patients, narcotics taken at six months were prescribed by outside providers. Significantly fewer patients used narcotics at six months for THA (15.4%) compared to TKA (52.7%) (*P* < 0.021). There was a significant difference in daily narcotic usage between patients who took narcotics preoperatively (22.9 MMEs) and opioid-naïve ones (13.4 MMEs) (*P* < 0.001). Provider efficiency scores were best at three weeks (76.6%) and three months (70%) but declined at six months (57.2%).

**Conclusions:**

Narcotic tapering practices were observed as postoperative daily narcotic intake decreased across six months. However, outside providers prescribed 80% of narcotics at six months, necessitating a better-coordinated practice with surgeons. Patients taking preoperative narcotics experienced higher daily MME requirements than their opioid-naïve counterparts. In terms of the percentage of patients on narcotics, THA is a better procedure for tapering patients off narcotics by six months.

**Supplementary Information:**

The online version contains supplementary material available at 10.1186/s42836-024-00276-w.

## Introduction

Total joint arthroplasty (TJA) is an effective treatment option for end-stage hip and knee osteoarthritis [1]. Despite the effectiveness of the procedure, immediate postoperative pain can be difficult to manage. An observational cohort study on 105 TJA patients conducted by *Wylde* et al. demonstrated that 58% of total knee arthroplasty (TKA) and 47% of total hip arthroplasty (THA) patients reported inadequate postoperative analgesia [2].

Historically, opioids have been the mainstay of acute TJA pain management [3, 4]. As a result, TJA surgeons are cautioned about the over-prescription of postoperative narcotics. Prescription drug monitoring programs (PDMPs) are an initiative at the state level to monitor, inform, and guide narcotic prescriptions [5]. PDMPs use morphine milligram equivalents (MMEs), which provide a common denominator to assess the dosage and frequency of narcotic usage [6, 7]. CDC guidelines caution prescribing narcotics with daily MMEs greater than 50 and avoiding daily MMEs greater than 90 [8, 9].

While these guidelines aid physicians in using PDMPs for prescribing narcotics, there are discrepancies regarding the education and interpretation of MMEs in PDMPs state-specific policies, leading to poor pain management [10]. In a prospective cohort study, *Tan* et al. found that physicians who were educated on MMEs failed to retain their knowledge long-term, which was a proposed cause of insufficient pain management [7].

Patients managed with at least 60 MMEs consistently in the preoperative setting were reported to have an 80% increased risk of consistent postoperative narcotic use, diminishing the intended effect in pain management [11]. In an observational retrospective study that included 100 TJA patients, *De Luca* et al. reported that in more than 50% of THA patients and more than 75% of TKA patients, postoperative analgesic regimens were ineffective when different postoperative pain protocols were used with varying daily MMEs [12]. The reported inadequacy of analgesia in both preoperative and postoperative periods necessitates a better system for evaluation and pain management.

The validated Detroit Interventional Pain Assessment (DIPA) scale is a pain scale that addresses these inadequacies and is validated in two parts. The first study validated the Interventional Pain Assessment (IPA) tool, a pain scale based on three pain assessment categories (0 = no pain, 1 = tolerable pain, 2 = intolerable pain). It was conducted on 322 ambulatory postoperative orthopedic patients in an outpatient setting [13]. It was used to guide treatment intervention for patients with a score of 0 or 1 indicating adequate pain management, not requiring further intervention, or a score of 2 signaling a change in treatment. The IPA scale was validated against a 0–10 numerical rating scale using a Kendall rank correlation. Results displayed a significant association with the NRS (τ = 0.58, *P* < 0.0001) and 82% of patients preferred the IPA scale over the NRS due to the simplicity and effectiveness in communicating patients’ pain.

**Fig. 1:**
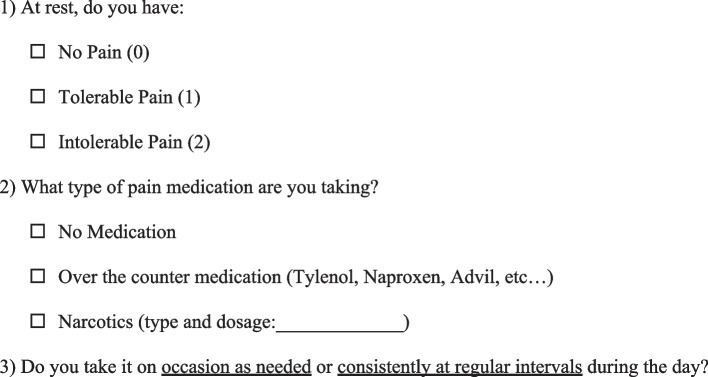
Detroit Interventional Pain Assessment Survey Total Joint Arthroplasty Cohort. Detroit Interventional Pain Assessment (DIPA) scale survey given verbally to patients. Questions consist of (1) pain assessment, (2) type of pain medication, and (3) frequency of use. A similar DIPA survey was used in a fracture cohort submitted to the Journal of Orthopaedic Trauma, but not yet published

The second study incorporated medication classes into the IPA scale and was called the Detroit Interventional Pain Assessment (DIPA) scale [14]. The DIPA scale was successfully applied to a cohort of 502 patients with trauma or arthroplasty, or after orthopedic surgery in an outpatient setting. In addition to the two non-narcotic medication classes (A: no pain medication, 0 MMEs; B: Over-the-counter pain medication, 0 MMEs), the number of narcotic medication classes was determined by a hierarchical cluster analysis with results displaying three groups. The MME ranges for these groups were established by a K-means cluster analysis, resulting in narcotic classes C (1–30 MMEs), D (31–79 MMEs), and E (80 + MMEs). The DIPA scale was used as an educational tool for orthopedic surgeons to classify medications into five main groups with associated daily MMEs using a questionnaire (Fig. 1) (Table 1). Providers were able to track their postoperative pain management progress and patients’ daily MMEs without complex interpretation at each postoperative clinic visit.

**Table 1:** Detroit Interventional Pain Assessment Scale Medication Classes

Class	Daily Morphine Milligram Equivalents (MMEs)	Types of Medications
A: No medication	0	N/A
B: Over-the-Counter	0	N/A
C: Occasional use of short-acting narcotics	1–30	schedule II and IV drugs (codeine-acetaminophen, tramadol, hydrocodone- acetaminophen, or oxycodone- acetaminophen)
D: Regular use of short-acting narcotics	31–79	schedule II drugs (hydrocodone-acetaminophen or oxycodone-acetaminophen)
E: Long-acting narcotics with breakthrough short-acting	80 +	schedule II drugs (hydromorphone, methadone, morphine, and oxycodone hydrochloride)

The purpose of this study was to use the DIPA scale to assess the type and quantity of narcotic medication prescribed after TJA and patient satisfaction with their pain management. The second objective was to compare these same post-operative pain control in Total Hip Arthroplasty (THA) versus Total Knee Arthroplasty (TKA) patients. We also wanted to compare patients with preoperative opioid usage to opioid-naïve patients. Finally, the last objective was to give the operating surgeons feedback on their pain management practice, patient satisfaction with their pain management, and possible areas for improvement.

## Methods

### Detroit interventional pain assessment scale

The DIPA scale rates patients’ pain as 0 (no pain), 1 (tolerable pain), or 2 (intolerable pain) and can be administered verbally or in writing (Fig. 1) [14]. Statistically validated medication classes with associated daily MMEs and corresponding types of narcotics are listed in Table 1.

Detroit Interventional Pain Assessment (DIPA) scale medication classification with associated daily morphine milligram equivalents (MMEs) and narcotic class. Class A represents no medication (0 MME); Class B over-the-counter (OTC) medications (0 MME); Class C occasional use of short-acting narcotics (1–30 MMEs, schedule II and IV drugs); Class D consistent use of short-acting narcotics (31–79 MMEs, schedule II drugs); Class E use of long-acting narcotics (80 + MMEs, schedule II drugs). This is the standard DIPA scale table that is used for all orthopedic patients, including a fracture cohort and was submitted to the Journal of Orthopedic Trauma, but not yet published.

Pain management progress is assessed by efficiency scores. These efficiency scores represent the percentage of patients experiencing no pain or tolerable pain in each postoperative period, with higher efficiency scores indicating better pain management.

$$\text{Efficiency scores }=\frac{\text{Sum of patients reporting no pain or tolerable pain in a specific postoperative period}}{\textrm{Total number of patients in the same postoperative period}}$$

A previous DIPA study reported that the best efficiency scores were between 75%–85% and occurred three weeks and three months after orthopedic surgery [14]. During these two postoperative periods, over 60% of patients were not using narcotics. However, when efficiency scores were between 60–70%, more patients reported intolerable pain both when not using opioids and on opioid regimens. An efficiency score of 70% or lower likely signaled improvement.

### Design

An IRB-approved prospective cohort study for immediate postoperative care was conducted at a tertiary care academic joint replacement practice for six months.

### Study sampling

Inclusion criteria included patients aged 18 and older who underwent primary THA and TKA presenting to the clinic during three scheduled follow-up visits over a period of six months. Exclusion criteria consisted of patients who underwent revision TJA procedure and those who had language or comprehension barriers.

### Joint clinic policy

Joint clinic policy on narcotics recommends providers should aim to stop narcotic prescriptions three months after the operation to decrease patients’ risk of prolonged narcotic usage.

## Procedure

All information pertinent to the patient was de-identified when patients arrived for their scheduled appointments. Interviews were verbally conducted by an independent observer at three weeks, three months, and six months postoperatively. All participants were asked questions based on the DIPA scale questionnaire regarding pain level at rest and daily baseline postoperative pain medication usage (Fig. 1). The Michigan PDMP, Michigan Automated Prescription System (MAPS) narcotics registry, was used to verify patients’ actual daily MMEs from narcotic prescriptions received. Each patient’s MAPS narcotics report was reviewed for their current clinic visit before prescribing another narcotic prescription and their daily MMEs from the report were recorded. Prescribed daily MMEs from the MAPS narcotics report and the DIPA scale-generated MME ranges based on patient-reported opioid usage were compared. Based on patients’ answers to the DIPA surveys, they were grouped according to their DIPA pain score and medication class.

Next, they were grouped according to their postoperative date (three weeks, three months, and six months).

**Fig. 2:**
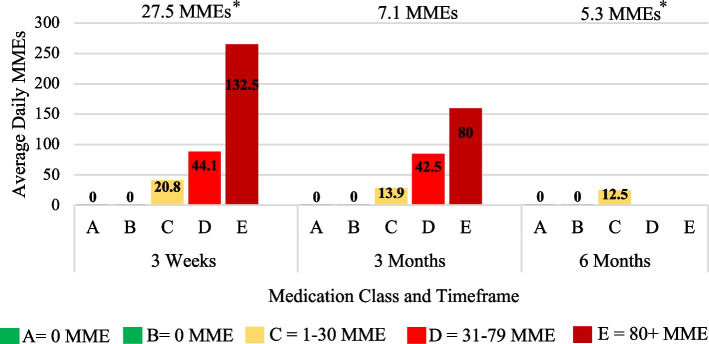
TJA Morphine Milligram Equivalents for Each Postoperative Period. Average daily morphine milligram equivalents (MMEs) for Total Joint Arthroplasty (TJA) at each postoperative period. Medication classes are represented as A no medication (green, 0 MME), B over-the-counter medication (green, 0 MME), C occasional use of short-acting narcotics (yellow, 1–30 MMEs), D consistent use of short-acting narcotics (red, 31–79 MMEs), and E long-acting narcotics (dark red, 80 + MMEs). The average daily MMEs for each medication class are displayed in the bars present at each postoperative period. The overall average for a specific postoperative period = (Sum of all daily MMEs for the specific postoperative period)/(total number of patients in same postoperative period). Statistical differences in MMEs from three weeks to three months are represented by an asterisk

A graph displaying the average daily MMEs for each DIPA medication class in every postoperative period was created (Fig. 2). The combined average daily MMEs for each postoperative period were also calculated and displayed at the top.

$$\text{Average daily MMEs for specific medication class }=\frac{\text{sum of each patients}{\prime}\text{daily MME for medication class in a postoperative period}}{\textrm{total number of patients in medication class in same postoperative period}}$$

**Fig. 3:**
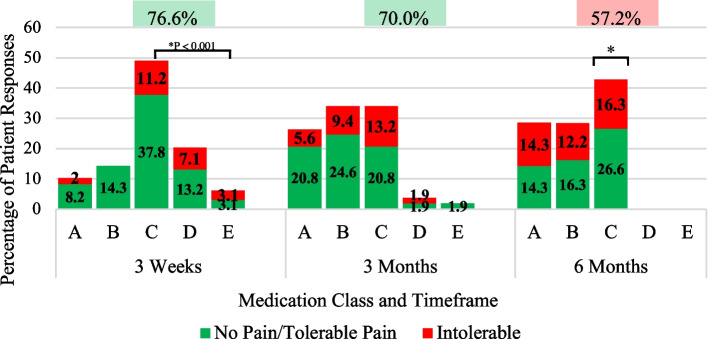
Total Joint Arthroplasty DIPA Graph. Detroit Interventional Pain Assessment (DIPA) graph for Total Joint Arthroplasty (TJA). Medication classes are represented as A (no medication, 0 MME), B (Over-the-counter medication, 0 MME), C (occasional use of short-acting narcotics, 1–30 MMEs), D (consistent use of short-acting narcotics, 31–79 MMEs), and E (long-acting narcotics). The percentage of patients in each medication class with their pain assessment (green = no pain/tolerable pain, red = intolerable pain) is displayed at three weeks, three months, and six months. Efficiency scores are represented as percentages displayed at each postoperative period. A statistical difference between the percentage of patients using narcotics from three weeks to six months is represented by an asterisk

**Fig. 4:**
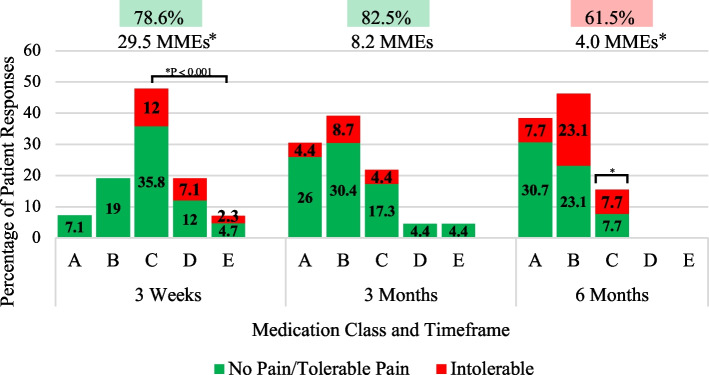
Total Hip Arthroplasty DIPA Graph. Detroit Interventional Pain Assessment (DIPA) graph for total hip arthroplasty (THA). Medication classes are represented as A (no medication, 0 Morphine Milligram Equivalent (MME)), B (Over-the-counter medication, 0 MME), C (occasional use of short-acting narcotics, 1–30 MMEs), D (consistent use of short-acting narcotics, 31–79 MMEs), and E (long-acting narcotics). The percentage of patients in each medication class with their pain assessment (green = no pain/tolerable pain, red = intolerable pain) is displayed at three weeks, three months, and six months. Efficiency scores are represented as percentages and average daily MMEs are displayed at each postoperative period. Statistical differences in MMEs and the percentage of patients using narcotics from three weeks to three months are represented by asterisks

**Fig. 5:**
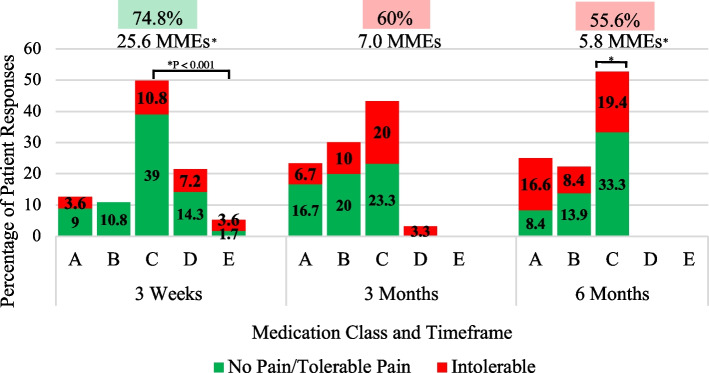
Total Knee Arthroplasty DIPA Graph. Detroit Interventional Pain Assessment (DIPA) graph for total knee arthroplasty (TKA). Medication classes are represented as A (no medication, 0 Morphine Milligram Equivalent (MME)), B (Over-the-counter medication, 0 MME), C (occasional use of short-acting narcotics, 1–30 MMEs), D (consistent use of short-acting narcotics, 31–79 MMEs), and E (long-acting narcotics). The percentage of patients in each medication class with their pain assessment (green = no pain/tolerable pain, red = intolerable pain) is displayed at three weeks, three months, and six months. Efficiency scores are represented as percentages and average daily MMEs are displayed at each postoperative period. Statistical differences in MMEs and the percentage of patients using narcotics from three weeks to three months are represented by asterisks

**Fig. 6:**
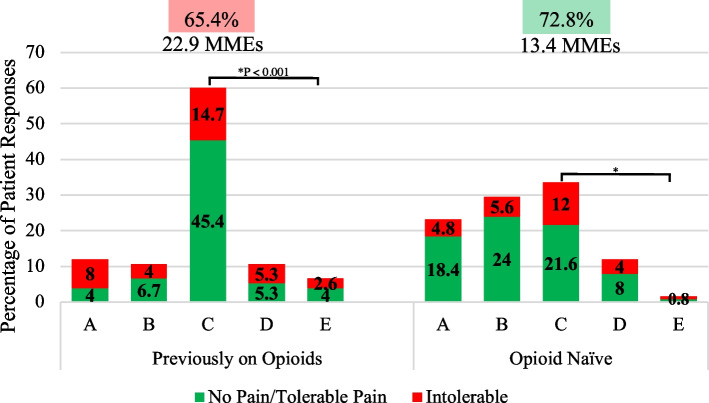
DIPA Graph for Patients Previously on Opioids and Opioid Naïve. Detroit Interventional Pain Assessment (DIPA) graph for patients previously on opioids and those who were opioid naïve. Medication classes are represented as A (no medication, 0 Morphine Milligram Equivalent (MME)), B (Over-the-counter medication, 0 MME), C (occasional use of short-acting narcotics, 1–30 MMEs), D (consistent use of short-acting narcotics, 31–79 MMEs), and E (long-acting narcotics). The percentage of patients in each medication class with their pain assessment (green = no pain/tolerable pain, red = intolerable pain) is displayed for the entire six-month study period. Efficiency scores are represented as percentages and average daily MMEs are displayed at each postoperative period. A statistical difference in the percentage of patients using narcotics between patients previously on opioids and opioid naïve patients is represented by an asterisk

A DIPA graph displaying patient categories of narcotic usage versus satisfaction was generated for the total cohort of patients and efficiency scores were displayed at the top for each postoperative period (Fig. 3). Patients were also separated into four groups (THA, TKA, Previously on Opioids, and Opioid Naive) and DIPA graphs were created for these groups with efficiency scores and daily MMEs for each postoperative period displayed at the top (Figs. 4, 5, and 6).

### Data analyses

All statistical analyses were conducted using the Statistical Package for the Social Sciences (SPSS Statistics v29; IBM Corp, Armonk, NY, USA) set at a significance level of 0.05. To validate Independent T-test assumptions, data were assessed with Shapiro–Wilk normality tests. However, due to non-normal distributions, Kruskal–Wallis tests were used to determine the differences at each postoperative period, the differences in the percentage of patients on narcotic usage at each postoperative period, and the differences in MMEs and percentage of patients on narcotics between THA and TKA patients and between patients previously on opioids and those who are opioid-naïve. A Mann–Whitney U test was performed to further test for specific differences between the two postoperative periods (Supplementary Information: Additional Files 1–8 for statistical results).

## Results

This study included 200 postoperative TJA patients. There were 56 (28%) males and 144 (72%) females, with an average age of 60.4 ± 9.9 years (range, 20 to 92) (Table 2). Of them, 121 patients (60.5%) underwent TKA, and 79 (39.5%) received THA. All efficiency scores and MME data were compared for patient demographics (Table 3).

**Table 2:** Demographic Characteristics of Patients

Demographics	Average	Total	
		***n***	**%**
**Gender**			
Female		144	72
Male		56	28
**Age**	60.4 ± 9.9		
**BMI**	36.8 ± 7.9		
BMI < 40		154	77
BMI > 40		46	23
Race/Ethnicity			
Black/African American		135	67.5
Not Specified		37	18.5
White/European		18	9
Other		10	5

Demographic information for patients consists of gender, age, body mass index (BMI), and race/ethnicity

**Table 3:** Demographic Characteristics of Patients and Efficiency Scores and Prescribed MMEs

Demographics	3 weeks			3 months			6 months		
	Efficiency Scores (IPA 0–1)			Efficiency Scores (IPA 0–1)			Efficiency Scores (IPA 0–1)		
	%	95% CI	*P*-value	%	95% CI	*P*-value	%	95% CI	*P*-value
Gender									
Female	80.0	69.8**–**89.8	0.258	70.2	54.5**–**82.8	0.913	53.8	37.5**–**69.4	0.567
Male	69.6	51.8**–**85.2		68.7	43.7**–**91.6		66.6	33.3**–**100	
BMI									
BMI < 40	78.6	68.2**–**87.9	0.370	73.9	60.7**–**87.0	0.198	56.2	38.4**–**72.7	1.000
BMI > 40	69.5	50.0**–**88.8		42.8	6.5**–**92.2		56.2	30.0**–**77.7	
Race/Ethnicity									
White/European	100		0.099	75.0	20.3**–**100	0.270	60.0	0**–**100	0.886
Black/African American	77.4	66**–**87.3		62.1	47.2**–**76.4		58.3	41.6**–**74.1	
Not Specified	62.5	42.3**–**82.1		87.5	57.1**–**100		40.0	0**–**100	
Other	100			100			100		
	3 weeks			3 months			6 months		
	AVG MMEs	95% CI	*P*-value	AVG MMEs	95% CI	*P*-value	AVG MMEs	95% CI	*P*-value
Gender									
Female	25.4	18.2**–**32.7	0.662	7.8	2.6**–**13.0	0.709	4.3	2.5**–**6.2	0.716
Male	31.6	17.7**–**45.4		8.9	1.2**–**16.5		7.2	0.36**–**15.2	
BMI									
BMI < 40	25.2	19.2**–**31.2	0.696	30.7	29.1**–**32.2	0.001	4.5	2.2**–**7.7	0.485
BMI > 40	35	14.1**–**55.9		47.2	42.7**–**51.7		5.4	2.5**–**8.8	
Race/Ethnicity									
White/European	13.8	4.3**–**23.4	0.184	0		0.263	10.5	0**–**25.1	0.786
Black/African American	30	20.3**–**39.7		9.9	5.1**–**15.9		4.4	2.4**–**6.3	
Not Specified	24.6	15.5**–**33.8		5.6	0**–**15.0		2.4	0**–**6.3	
Other	40	18.4**–**61.5		5	0**–**15.0		5.0	0**–**10.0	

Demographic characteristics of patients (gender, age, BMI, and race/ethnicity) and corresponding efficiency scores and prescribed MMEs at three weeks, three months, and six months after operation. Efficiency scores were calculated as the percentage of patients reporting no pain or tolerable pain in a specific postoperative period

### Narcotic usage and efficiency scores in total joint arthroplasty

After having verified all patients’ daily MMEs via the MAPS narcotics registry, the DIPA scale medication classes correctly reported 100% of patients’ actual daily MMEs represented by their current pain medication regimens. The surveyed TJA patients displayed a daily average narcotic intake of 27.5 MMEs (two weeks), 7.1 MMEs (three months), and 5.3 MMEs (six months). Patients achieved improvement in their overall opioid consumption, using only intermittent short-acting narcotics at six months (Fig. 2). This was represented by a significant decrease in the overall average daily MMEs from three weeks (27.5 MMEs, 95% CI 21.3–34.4) to six months (5.3 MMEs, 95% CI 3.2–7.7) (*P* < 0.001).

Efficiency scores representing patients’ satisfaction with pain medication regimens revealed the best pain management practices at three weeks (76.6%) and three months (70.0%) (Fig. 3). At three weeks, 24.5% of patients’ medication regimens consisted of no medication or over-the-counter (OTC) pain medication (Classes A and B), with 2% reporting intolerable pain. The remaining 75.5% of patients at three weeks reported using narcotics, mostly short-acting narcotics as needed (Class C), with 21.4% reporting inadequacy with narcotic regimens. When tracking the origin of narcotic prescriptions, 61% of narcotic prescriptions were leftover from surgical discharge (26.7 ± 15.3 MMEs), 19.1% originated from providers in the practice (29.8 ± 12.5 MMEs), and 19.1% came from outside providers (91 ± 62.2 MMEs).

By three months, 60.4% of patients’ medication regimens didn’t include narcotics (Classes A and B), with 15% reporting intolerable pain. Of those patients who used narcotics (Classes C, D, and E) for pain management at three months (39.7%), 15.1% also reported inadequacy. Fifty percent of narcotic prescriptions were from outside providers (26.2 ± 23.6MMEs), 45% originated from providers in the practice (14.4 ± 7.9 MMEs), and 5% were left over from surgical discharge (10.0 ± 0.0 MMEs).

Patients presenting to the clinic at six months postoperatively were not satisfied with their pain management, as evidenced by a 57.2% efficiency score. Among the patients reporting intolerable pain at six months in classes A, B, and C, 57.1% of them were previously on narcotics. Eighty percent of patients reported receiving narcotic prescriptions from outside providers at six months. All patients who were taking narcotics (Class C) and reporting intolerable pain at six months underwent previous orthopedic surgeries and had histories of orthopedic conditions, such as osteoarthritis in other joints, bursitis, spinal stenosis, sciatica, and spondylolisthesis. While the six-month postoperative period had the lowest efficiency score, there was a significant decrease in the percentage of patients taking narcotics from three weeks (75.5%, 95% CI 68.2–85.0) to six months (42.9%, 95% CI 27.4–56.5) (*P* < 0.001).

### THA narcotic usage and efficiency scores

When patients were stratified by types of arthroplasty procedures, 73.9% of patients were taking narcotics (Classes C, D, and E) at three weeks for THA (Fig. 4). At three months, there was a 58.7% decrease in narcotic usage and a 79.1% decrease by six months. A significant decrease in the percentage of patients on narcotics was also observed from three weeks (73.9%, 95% CI 62.7–88.3) to six months (15.4%, 95% CI 0.0–38.4) (*P* < 0.001). There was a significant difference in the average daily MMEs between three weeks (29.5 MMEs, 95% CI 19.2–42.0) and six months (4.0 MMEs, 95% CI 0.0–10.7) (*P* < 0.001). THA demonstrated greater patient satisfaction efficiency scores, predominantly at three weeks (78.6%) and three months (82.5%) after operation. While the efficiency score for THA patients at six months postoperative (61.5%) was the highest in all groups (total patients and TKA) at that specific time, pain management during that time needed improvement.

### TKA narcotic usage and efficiency scores

Analysis showed that, in TKA patients, there was a significant difference in the percentage of patients on narcotics between three weeks (76.6%, 95% CI 63.7–85.9) and six months (52.7%, 95% CI 35.8–69.2) (*P* < 0.029) (Fig. 5). As patients’ postoperative rehabilitation progressed, pain medications transitioned from narcotic usage towards non-steroid anti-inflammatory medications and acetaminophen, or no medications. This was supported by the significant difference in the decrease in daily MMEs between three weeks (25.6 MMEs, 95% CI 18.1–34.7) and six months (5.8 MMEs, 95% CI 3.4–8.5) (*P* < 0.001). Efficiency scores were best at three weeks, representing 74.8% patient satisfaction, but needed improvement at three months (60%) and six months (55.6%).

### Differences in the narcotic usage and the efficiency score between THA and TKA

No significant difference was found in daily MMEs at each postoperative period (*P* = 0.847) between THA and TKA patients. However, the percentage of THA patients on narcotics at six months (15.4%, 95% CI 0.0–37.4) was significantly less than that of TKA patients (52.7%, 95% CI 36.5–70.5) (*P* = 0.021). When THA and TKA patients were compared, better efficiency scores were observed in THA patients.

### Patients previously on narcotics vs. their opioid-naïve counterparts

Thirty-four percent of patients with a previous history of narcotic use within two years of TJA reported intolerable pain during this study (Fig. 6). There was a significant difference in overall average daily MMEs between patients who previously took narcotics preoperatively (22.9 MMEs, 95% CI 16.2–30.2) and those who did not (13.4 MMEs, 95% CI 9.7–17.7) (*P* < 0.001). While no significant difference was found in the percentage of patients reporting tolerable pain between these two groups (*P* = 0.323), the opioid-naïve group had 7.4% more patients reporting tolerable pain (72.8%, 95% CI 63.1–79.7) than those who previously took narcotics in the past two years (65.4%, 95% CI 53.7–76.3).

## Discussion

Opiate medication is the mainstay of pain relief for TJA, but prescription practices can potentially contribute to opioid adverse events [15, 16]. In recent studies, results focused on guidelines for prescribing a specific number of pills (30–40 pills of Oxycodone at 5 mg) for TJA, rather than educating surgeons on their pain management practice [17, 18]. Without the proper education on pain management and MMEs, physician error in MME interpretation and over-prescription of narcotics becomes more plausible [19].

A previous DIPA methodological study educated surgeons on daily MMEs and, by using the DIPA scale, displayed pain management shortcomings across a postoperative period [14]. Results suggested that providers should be more cognizant of their pain management at two weeks since 62% of patients reported experiencing intolerable pain. This current study sought to identify the impact of the DIPA scale in TJA with application in a prospective cohort study.

Surgeons in this study were efficient at tapering opioids throughout the six-month postoperative period, evidenced by significant decreases in the percentage of patients on narcotics. However, when patients presented to the clinic at six months, surgeons were inefficient at managing patients’ postoperative pain. All patients taking narcotics and experiencing inadequate pain management at six months had a history of previous orthopedic surgery and chronic orthopedic conditions, such as osteoarthritis in other joints, bursitis, spinal stenosis, sciatica, or spondylolisthesis, suggesting that these are possible risk factors of increasing postoperative TJA pain and prolonged narcotic usage [20]. When efficiency scores were presented to the surgeons, they suggested implementing new follow-up visits at nine weeks and one year to better monitor patients’ postoperative progress and pain management.

The patients who were surveyed had chronic arthritis that progressed to the end stage in the joint that required surgical intervention. In many of the cases, arthritis may have been present in other joints, causing centralized pain and, subsequently, affecting their postoperative pain management [21, 22]. Because of the possibility of arthritis being present in multiple joints, patients were more susceptible to taking preoperative narcotics, affecting the efficacy of postoperative analgesic regimens. Patients experiencing intolerable pain at six months may need other treatment modalities, such as an additional round of physical therapy to strengthen surrounding muscles or visiting a pain medicine physician to find adequate analgesic regimens [23]. Additionally, patients may need to be referred to another orthopedic specialist if they are experiencing pain in areas other than the hip or knee joints.

Over a third of patients who had reported taking narcotics previously reported experiencing intolerable pain postoperatively and had significantly higher daily MMEs. This indicates that patients who previously took narcotics before receiving surgery are more likely to ingest stronger narcotics or take them more frequently postoperatively. Patients taking narcotics prior to TJA should be closely monitored throughout the postoperative rehabilitation period to prevent the possibility of protracted narcotic usage.

Despite surgeons routinely stopping narcotic prescriptions at three months, 80% of narcotic prescriptions at six months were from outside providers, suggesting the necessity of coordinated efforts in pain management between TJA surgeons and outside providers in the preoperative and postoperative periods to prevent prolonged opioid use. While surgeons were effective at tapering postoperative opioids, some patients may need to return to their baseline narcotic usage (preoperative dosage) to achieve adequate pain management.

Similar daily narcotic intakes were observed in both THA and TKA resulting in no significant difference in daily MMEs at each postoperative period (*P*= 0.847). This indicates that both groups were managed with similar narcotic dosages. While patients were prescribed similar medications and both procedures are intended to relieve pain and restore function, THA patients exhibited better pain scores at every postoperative period. This, very likely, reflects that THA is a better pain-relieving procedure than TKA [24]. Additionally, when comparing the percentage of patients on narcotics, better opioid tapering practices were observed in THA, reflecting that THA is a better procedure for tapering patients off narcotics by six months. The increase in TKA patients using narcotics at six months may mirror the prescription of inadequate narcotic dosages during the previous postoperative period (three months), which limits patients’ ambulation and their ability to complete physical therapy exercises for improving strength and range of motion. The reduction in ambulation during this time could potentially increase patients’ pain sensitivity [25]. As a result, surgeons may need to prescribe higher dosages of narcotics at previous postoperative periods for TKA patients, possibly 10 MMEs/day at three months, or refer TKA patients with uncontrolled pain to pain management specialists.

In addition to tapering opioids, current research on minimizing opioid prescriptions after TJA is aimed at implementing postoperative pain control protocols [4]. Woelber et al. conducted a retrospective study on 40 TKA patients. Patients were compared prior to the Minimizing Opioids After Joint Operation (MOJO) postoperative pain protocol and post-MOJO. Their methods included preoperative physical rehabilitation and patient education on daily MMEs. They found a significant difference in daily MMEs (82 MMEs and 31 MMEs, *P* < 0.01) and reported pain (5.5 and 4.1, *P* = 0.01) between the pre-MOJO and post-MOJO groups. Additionally, post-MOJO pain regimens consisted of taking short-acting narcotics as needed, with higher doses of non-opioids and muscle relaxants taken consistently throughout the postoperative period.

The MOJO postoperative pain control protocol has similarities to the DIPA scale, with the most important matters being patient and provider education on daily MMEs and the reduction in daily MMEs during the postoperative period. The DIPA scale has proven its effectiveness at addressing shortcomings in TJA pain management by prospectively following patients over time through adequate pain assessment and offering surgeons feedback on their prescribing tendencies.

The beauty of the DIPA scale in clinical practice is in its simplicity. The three straightforward questions enable clear and concise communication of the patient’s pain level and medication use. The alphabetical medication classes provide both TJA surgeons and patients with a standard education on daily MMEs and corresponding medications which can help align treatment expectations and practices. The DIPA scale offers reduced interpretation variability as well as guidance for prescribing, which together can help minimize physician error from over- or under-medication. The DIPA scale also provides enhanced patient awareness and engagement. Different from the standard numerical rating scale, by having patients present the type and frequency of pain medication, the DIPA scale helps patients better understand their medication regimens and their impact on their pain management. This awareness can empower patients to make informed decisions about their pain management strategies and improve adherence to treatments. Regular use of the DIPA scale during follow-up visits ensures that there is ongoing assessment and adjustment of pain management strategies. This overall enhances the quality of care provided to TJA patients. Future work will focus on the DIPA scale in TJA clinics to suggest possible combinations of treatment modalities and analgesia that yield the most successful postoperative pain management, or tapering practices that lead to better patient satisfaction.

This study was subjected to some limitations. The small sample size of 200 patients limits the generalizability to a large population of TJA patients. While narcotic usage was a part of the primary outcome measures, other alternative forms of pain treatment were not explored, which could potentially affect providers’ pain management efficiency scores. For patients taking narcotics prior to TJA, their daily baseline narcotic usage before TJA was not recorded. This was a potential confounding variable that could affect patients’ postoperative narcotic usage and subsequent pain management. While patients’ medical histories were recorded, they were not separated from the conditions that caused them to have TJA and these conditions were not compared in terms of pain management or opioid usage. Furthermore, there were two surgeons who performed the procedures for our included patients. Variations in technique, differences between robotic and non-robotic operations as well as implant vendors may play a role in patient pain levels postoperatively. These factors were not compared in our study.

## Conclusion

The DIPA graph for TJA patients displayed opioid tapering across six months. However, better pain management was necessitated when patients presented to the clinic at six months postoperatively, with intolerable pain being likely related to chronic orthopedic conditions, such as osteoarthritis in other joints and spinal stenosis. Providers suggested following up with patients, especially those with chronic orthopedic conditions at nine weeks and one year. The comparison showed that, in terms of the percentage of patients on narcotics, THA is a better procedure for tapering patients off narcotics by six months. Patients who reported taking preoperative narcotics suffered from more intolerable pain and significantly higher daily MMEs postoperatively. Coordinated pain management efforts between TJA surgeons and outside providers during the preoperative and postoperative periods are necessitated for adequate pain management and to prevent prolonged narcotic usage. Future studies should be aimed at developing a set of guidelines for TJA aftercare involving education on daily MMEs via the DIPA scale, providing a daily MME range, or tapering practices that lead to better patient satisfaction.

## Supplementary Information

Supplementary Material 1.

Supplementary Material 2.

Supplementary Material 3.

Supplementary Material 4.

Supplementary Material 5.

Supplementary Material 6.

Supplementary Material 7.

Supplementary Material 8.

## Data Availability

The datasets used and/or analyzed during the current study are available from the corresponding author on reasonable request.
