# Supplementary material for: Assessing pain management in total joint arthroplasty using the Detroit interventional pain assessment scale—A prospective cohort study
Source: Arthroplasty. 2024 Nov 1;6:55. doi: 10.1186/s42836-024-00276-w (PMC11529018; doi:10.1186/s42836-024-00276-w)
Supplement: Supplementary file 1 — Supplementary Material 1. [file 42836_2024_276_MOESM1_ESM.pdf]

TJA difference in MMEs

Case Processing Summary

		Cases					
		Valid		Missing		Total	
	Time	N	Percent	N	Percent	N	Percent
MME	3 weeks	98	100.0%	0	0.0%	98	100.0%
	6 months	49	100.0%	0	0.0%	49	100.0%

Descriptives

Bootstrap Specifications

Sampling Method	Simple
Number of Samples	1000
Confidence Interval Level	95.0%
Confidence Interval Type	Percentile

						Bootstrap[a]		
	Time			Statistic	Std. Error	Bias	Std. Error	95% Confidence Interval Lower
MME	3 weeks	Mean		27.5531	3.31201	-.0306	3.2434	21.3814
		95% Confidence Interval for Mean	Lower Bound	20.9796				
			Upper Bound	34.1265				
		5% Trimmed Mean		22.7234		.3407	2.6126	18.3216
		Median		22.5000		.6482	4.7948	15.0000
		Variance		1075.000		-12.922	361.515	441.857
		Std. Deviation		32.78719		-.68130	5.59649	21.02039
		Minimum		.00				
		Maximum		180.00				
		Range		180.00				
		Interquartile Range		26.92		2.54	5.22	20.00
		Skewness		2.744	.244	-.160	.478	1.501
		Kurtosis		9.585	.483	-.557	3.502	3.087
	6 months	Mean		5.3810	1.15965	.0048	1.1677	3.2560
		95%	Lower	3.0494				

	Confidence Interval for Mean	Bound					
		Upper Bound	7.7126				
	5% Trimmed Mean		4.3860		.0897	1.1987	2.3489
	Median		.0000		.6021	1.5802	.0000
	Variance		65.894		-.889	18.572	32.293
	Std. Deviation		8.11753		-.13951	1.16529	5.68265
	Minimum		.00				
	Maximum		30.00				
	Range		30.00				
	Interquartile Range		10.00		-.48	1.65	5.00
	Skewness		1.654	.340	-.037	.360	.996
	Kurtosis		2.212	.668	.010	1.697	-.210

Descriptives

	Time			Bootstrap 95% Confidence Interval Upper
MME	3 months	Mean		34.4883
		95% Confidence Interval for Mean	Lower Bound	
			Upper Bound	
		5% Trimmed Mean		28.8603
		Median		30.0000
		Variance		1844.650
		Std. Deviation		42.94938
		Minimum		
		Maximum		
		Range		
		Interquartile Range		40.00
		Skewness		3.415
		Kurtosis		16.763
	6 months	Mean		7.7531
		95% Confidence Interval for Mean	Lower Bound	
			Upper Bound	
		5% Trimmed Mean		6.9481
		Median		7.5000
		Variance		104.158

	Std. Deviation	10.20580
	Minimum	
	Maximum	
	Range	
	Interquartile Range	10.98
	Skewness	2.466
	Kurtosis	6.486

Tests of Normality

		Kolmogorov-Smirnov[a]			Shapiro-Wilk		
	Time	Statistic	df	Sig.	Statistic	df	Sig.
MME	3 weeks	.205	98	<.001	.707	98	<.001
	6 months	.318	49	<.001	.705	49	<.001

a. Lilliefors Significance Correction

Test of Homogeneity of Variance

		Levene Statistic	df1	df2	Sig.
MME	Based on Mean	14.900	1	145	<.001
	Based on Median	15.104	1	145	<.001
	Based on Median and with adjusted df	15.104	1	105.967	<.001
	Based on trimmed mean	13.978	1	145	<.001

Kruskal-Wallis Test

Ranks

	Time	N	Mean Rank
MME	3 months	98	87.77
	6 months	49	46.46
	Total	147	

Test Statistics[a,b]

	MME
Kruskal-Wallis H	32.256
df	1
Asymp. Sig.	<.001

a. Kruskal Wallis Test

b. Grouping Variable: Time

Mann-Whitney Test

Ranks

	Time	N	Mean Rank	Sum of Ranks
MME	3 weeks	98	87.77	8601.50
	6 months	49	46.46	2276.50
	Total	147		

Test Statistics[a]

	MME
Mann-Whitney U	1051.500
Wilcoxon W	2276.500
Z	-5.679
Asymp. Sig. (2-tailed)	<.001

a. Grouping Variable: Time
