# Supplementary material for: Assessing pain management in total joint arthroplasty using the Detroit interventional pain assessment scale—A prospective cohort study
Source: Arthroplasty. 2024 Nov 1;6:55. doi: 10.1186/s42836-024-00276-w (PMC11529018; doi:10.1186/s42836-024-00276-w)
Supplement: Supplementary file 2 — Supplementary Material 2. [file 42836_2024_276_MOESM2_ESM.pdf]

TJA difference in percentage of patients on narcotics

Time

Case Processing Summary

		Cases					
		Valid		Missing		Total	
	Time	N	Percent	N	Percent	N	Percent
Percentage_Patients	3 weeks	98	100.0%	0	0.0%	98	100.0%
	6 months	49	100.0%	0	0.0%	49	100.0%

Bootstrap

Bootstrap Specifications

Sampling Method	Simple
Number of Samples	1000
Confidence Interval Level	95.0%
Confidence Interval Type	Percentile

Descriptives

						Bootstrap[a]	
	Time			Statistic	Std. Error	Bias	Std. Error
Percentage_Patients	3 weeks	Mean		76.5306	4.30311	.0686	4.2637
		95% Confidence Interval for Mean	Lower Bound	67.9901			
			Upper Bound	85.0711			
		5% Trimmed Mean		79.4785		.0762	4.7374
		Median		100.0000		.0000	.0000
		Variance		1814.643		-21.967	229.326
		Std. Deviation		42.59863		-.34894	2.76551
		Minimum		.00			
		Maximum		100.00			

		Range		100.00			
		Interquartile Range		.00		37.47	46.90
		Skewness		-1.272	.244	-.034	.301
		Kurtosis		-.392	.483	.180	.864
	6 months	Mean		42.8571	7.14286	-.5847	7.2337
		95% Confidence Interval for Mean	Lower Bound	28.4955			
			Upper Bound	57.2188			
		5% Trimmed Mean		42.0635		-.6497	8.0374
		Median		.0000		13.9000	33.2851
		Variance		2500.000		-61.417	136.096
		Std. Deviation		50.00000		-.63837	1.41920
		Minimum		.00			
		Maximum		100.00			
		Range		100.00			
		Interquartile Range		100.00		-.72	7.84
		Skewness		.298	.340	.037	.322
		Kurtosis		-1.994	.668	.131	.297

Descriptives

	Time			Bootstrap 95% Confidence Interval Lower	Bootstrap 95% Confidence Interval Upper
Percentage_Patients	3 weeks	Mean		68.2704	85.0560
		95% Confidence Interval for Mean	Lower Bound		
			Upper Bound		
		5% Trimmed Mean		70.3005	88.9511
		Median		100.0000	100.0000
		Variance		1285.806	2187.238
		Std. Deviation		35.85814	46.76792
		Minimum			
		Maximum			

		Range			
		Interquartile Range		.00	100.00
		Skewness		-2.001	-.797
		Kurtosis		-1.393	2.052
	6 months	Mean		27.4593	56.5217
		95% Confidence Interval for Mean	Lower Bound		
			Upper Bound		
		5% Trimmed Mean		24.9548	57.2464
		Median		.0000	100.0000
		Variance		2031.694	2560.455
		Std. Deviation		45.07431	50.60094
		Minimum			
		Maximum			
		Range			
		Interquartile Range		100.00	100.00
		Skewness		-.272	1.041
		Kurtosis		-2.102	-.955

a. Unless otherwise noted, bootstrap results are based on 1000 bootstrap samples

Tests of Normality

		Kolmogorov-Smirnov[a]			Shapiro-Wilk		
	Time	Statistic	df	Sig.	Statistic	df	Sig.
Percentage_Patients	3 weeks	.474	98	<.001	.525	98	<.001
	6 months	.376	49	<.001	.629	49	<.001

a. Lilliefors Significance Correction

Test of Homogeneity of Variance

		Levene Statistic	df1	df2	Sig.
Percentage_Patients	Based on Mean	15.527	1	145	<.001
	Based on Median	6.015	1	145	.015
	Based on Median and with adjusted df	6.015	1	141.469	.015
	Based on trimmed mean	15.527	1	145	<.001

Kruskal-Wallis Test

Ranks

	Time	N	Mean Rank
Percentage_Patients	3 weeks	98	82.25
	6 months	49	57.50
	Total	147	

Test Statistics[a,b]

	Percentage_Patients
Kruskal-Wallis H	16.237
df	1
Asymp. Sig.	<.001

a. Kruskal Wallis Test
b. Grouping Variable: Time

Mann-Whitney Test

Ranks

	Time	N	Mean Rank	Sum of Ranks
Percentage_Patients	3 weeks	98	82.25	8060.50
	6 months	49	57.50	2817.50
	Total	147		

Test Statistics[a]

	Percentage_Patients
Mann-Whitney U	1592.500
Wilcoxon W	2817.500
Z	-4.030
Asymp. Sig. (2-tailed)	<.001

a. Grouping Variable: Time
