# Supplementary material for: Assessing pain management in total joint arthroplasty using the Detroit interventional pain assessment scale—A prospective cohort study
Source: Arthroplasty. 2024 Nov 1;6:55. doi: 10.1186/s42836-024-00276-w (PMC11529018; doi:10.1186/s42836-024-00276-w)
Supplement: Supplementary file 3 — Supplementary Material 3. [file 42836_2024_276_MOESM3_ESM.pdf]

THA difference in MMEs

Time

Case Processing Summary

	Time	Cases					
		Valid		Missing		Total	
		N	Percent	N	Percent	N	Percent
MME	1.00	42	100.0%	0	0.0%	42	100.0%
	2.00	13	100.0%	0	0.0%	13	100.0%

Bootstrap Specifications

Sampling Method	Simple
Number of Samples	1000
Confidence Interval Level	95.0%
Confidence Interval Type	Percentile

Descriptives

	Time			Statistic	Std. Error	Bootstrap[a] Bias	Bootstrap[a] Std. Error	Bootstrap[a] 95% Confidence Interval Lower
MME	3 weeks	Mean		29.5857	5.72302	.2291	5.6747	19.2227
		95% Confidence Interval for Mean	Lower Bound	18.0278				
			Upper Bound	41.1436				
		5% Trimmed Mean		24.2222		.9755	5.1726	16.5049
		Median		22.5000		.7817	6.2356	11.3000
		Variance		1375.626		-11.569	601.873	294.713
		Std. Deviation		37.08943		-1.14369	8.48721	17.16719
		Minimum		.00				
		Maximum		180.00				

		Range		180.00				
		Interquartile Range		28.47		1.74	7.02	19.70
		Skewness		2.545	.365	-.210	.651	.544
		Kurtosis		7.402	.717	-.534	4.372	-.417
	6 months	Mean		4.0592	2.77843	.0267	2.8333	.0000
		95% Confidence Interval for Mean	Lower Bound	-1.9944				
			Upper Bound	10.1129				
		5% Trimmed Mean		2.8436		.3488	2.8287	.0000
		Median		.0000		.0797	1.0777	.0000
		Variance		100.356		-7.824	56.262	.000
		Std. Deviation		10.01776		-1.27342	4.01049	.00000
		Minimum		.00				
		Maximum		30.00				
		Range		30.00				
		Interquartile Range		.00		5.56	9.52	.00
		Skewness		2.280	.616	-.018[b]	.988[b]	.555[b]
		Kurtosis		3.991	1.191	.936[b]	5.524[b]	-2.030[b]

Descriptives

	Time			Bootstrap 95% Confidence Interval Upper
MME	3 weeks	Mean		42.0400
		95% Confidence Interval for Mean	Lower Bound	
			Upper Bound	
		5% Trimmed Mean		36.7517
		Median		30.0000
		Variance		2606.728
		Std. Deviation		51.05612
		Minimum		
		Maximum		
		Range		
		Interquartile Range		45.00
		Skewness		3.649
		Kurtosis		18.272
	6 months	Mean		10.7854
		95% Confidence Interval for Mean	Lower Bound	
			Upper Bound	

	5% Trimmed Mean	10.3171
	Median	.0000
	Variance	197.802
	Std. Deviation	14.06422
	Minimum	
	Maximum	
	Range	
	Interquartile Range	30.00
	Skewness	4.123[b]
	Kurtosis	17.000[b]

a. Unless otherwise noted, bootstrap results are based on 1000 bootstrap samples
b. Based on 869 samples

Tests of Normality

		Kolmogorov-Smirnov[a]			Shapiro-Wilk		
	Time	Statistic	df	Sig.	Statistic	df	Sig.
MME	3 weeks	.234	42	<.001	.702	42	<.001
	6 months	.503	13	<.001	.464	13	<.001

a. Lilliefors Significance Correction

Test of Homogeneity of Variance

		Levene Statistic	df1	df2	Sig.
MME	Based on Mean	3.742	1	53	.058
	Based on Median	4.567	1	53	.037
	Based on Median and with adjusted df	4.567	1	43.524	.038
	Based on trimmed mean	3.824	1	53	.056

Kruskal-Wallis Test

Ranks

	Time	N	Mean Rank
MME	3 weeks	42	32.04
	6 months	13	14.96

	Total	55	

Test Statistics[a,b]

	MME
Kruskal-Wallis H	12.024
df	1
Asymp. Sig.	<.001

a. Kruskal Wallis Test
b. Grouping Variable: Time

Mann-Whitney Test

Ranks

	Time	N	Mean Rank	Sum of Ranks
MME	3 weeks	42	32.04	1345.50
	6 months	13	14.96	194.50
	Total	55		

Test Statistics[a]

	MME
Mann-Whitney U	103.500
Wilcoxon W	194.500
Z	-3.468
Asymp. Sig. (2-tailed)	<.001

a. Grouping Variable: Time
