# Supplementary material for: Assessing pain management in total joint arthroplasty using the Detroit interventional pain assessment scale—A prospective cohort study
Source: Arthroplasty. 2024 Nov 1;6:55. doi: 10.1186/s42836-024-00276-w (PMC11529018; doi:10.1186/s42836-024-00276-w)
Supplement: Supplementary file 4 — Supplementary Material 4. [file 42836_2024_276_MOESM4_ESM.pdf]

THA significant difference in percentage of patients on narcotics

Time

Case Processing Summary

		Cases					
		Valid		Missing		Total	
	Time	N	Percent	N	Percent	N	Percent
Percentage_Patients	3 weeks	42	100.0%	0	0.0%	42	100.0%
	6 months	13	100.0%	0	0.0%	13	100.0%

Bootstrap Specifications

Sampling Method	Simple
Number of Samples	1000
Confidence Interval Level	95.0%
Confidence Interval Type	Percentile

Time

Descriptives

						Bootstrap[a]	
	Time			Statistic	Std. Error	Bias	Std. Error
Percentage_Patients	3 weeks	Mean		76.1905	6.65172	-.1782	6.4642
		95% Confidence Interval for Mean	Lower Bound	62.7571			
			Upper Bound	89.6239			
		5% Trimmed Mean		79.1005		-.2010	7.1731
		Median		100.0000		.0000	.0000
		Variance		1858.304		-32.857	344.564
		Std. Deviation		43.10805		-.59295	4.23447
		Minimum		.00			

		Maximum		100.00			
		Range		100.00			
		Interquartile Range		25.00		21.75	47.29
		Skewness		-1.276	.365	-.059	.501
		Kurtosis		-.393	.717	.425	2.044
	6 months	Mean		15.3846	10.41543	-.0676	10.6983
		95% Confidence Interval for Mean	Lower Bound	-7.3087			
			Upper Bound	38.0779			
		5% Trimmed Mean		11.5385		.6879	10.9074
		Median		.0000		.5500	7.2284
		Variance		1410.256		-123.496	749.229
		Std. Deviation		37.55338		-4.91298	14.88579
		Minimum		.00			
		Maximum		100.00			
		Range		100.00			
		Interquartile Range		.00		22.65	38.35
		Skewness		2.179	.616	.057[b]	1.039[b]
		Kurtosis		3.223	1.191	1.571[b]	5.831[b]

Descriptives

				Bootstrap 95% Confidence Interval	
	Time			Lower	Upper
Percentage_Patients	3 weeks	Mean		62.7907	88.3721
		95% Confidence Interval for Mean	Lower Bound		
			Upper Bound		
		5% Trimmed Mean		64.2119	92.6357
		Median		100.0000	100.0000
		Variance		1052.049	2393.577
		Std. Deviation		32.43530	48.92419
		Minimum			
		Maximum			
		Range			
		Interquartile Range		.00	100.00
		Skewness		-2.481	-.549
		Kurtosis		-1.792	4.359
	6 months	Mean		.0000	38.4615

	95% Confidence Interval for Mean	Lower Bound		
		Upper Bound		
	5% Trimmed Mean		.0000	37.1795
	Median		.0000	.0000
	Variance		.000	2571.429
	Std. Deviation		.00000	50.70926
	Minimum			
	Maximum			
	Range			
	Interquartile Range		.00	100.00
	Skewness		.493[b]	4.123[b]
	Kurtosis		-2.094[b]	17.000[b]

a. Unless otherwise noted, bootstrap results are based on 1000 bootstrap samples
b. Based on 863 samples

Tests of Normality

		Kolmogorov-Smirnov[a]			Shapiro-Wilk		
	Time	Statistic	df	Sig.	Statistic	df	Sig.
Percentage_Patients	3 weeks	.472	42	<.001	.529	42	<.001
	6 months	.505	13	<.001	.446	13	<.001

a. Lilliefors Significance Correction

Test of Homogeneity of Variance

		Levene Statistic	df1	df2	Sig.
Percentage_Patients	Based on Mean	1.903	1	53	.173
	Based on Median	.401	1	53	.529
	Based on Median and with adjusted df	.401	1	52.403	.529
	Based on trimmed mean	1.903	1	53	.173

Kruskal-Wallis Test

Ranks

	Time	N	Mean Rank
Percentage_Patients	3 weeks	42	31.95
	6 months	13	15.23
	Total	55	

Test Statistics[a,b]

	Percentage_Patients
Kruskal-Wallis H	15.268
df	1
Asymp. Sig.	<.001

a. Kruskal Wallis Test

b. Grouping Variable: Time

Mann-Whitney Test

Ranks

	Time	N	Mean Rank	Sum of Ranks
Percentage_Patients	3 weeks	42	31.95	1342.00
	6 months	13	15.23	198.00
	Total	55		

Test Statistics[a]

	Percentage_Patients
Mann-Whitney U	107.000
Wilcoxon W	198.000
Z	-3.907
Asymp. Sig. (2-tailed)	<.001

a. Grouping Variable: Time
