# Supplementary material for: Assessing pain management in total joint arthroplasty using the Detroit interventional pain assessment scale—A prospective cohort study
Source: Arthroplasty. 2024 Nov 1;6:55. doi: 10.1186/s42836-024-00276-w (PMC11529018; doi:10.1186/s42836-024-00276-w)
Supplement: Supplementary file 5 — Supplementary Material 5. [file 42836_2024_276_MOESM5_ESM.pdf]

TKA difference in MMEs

Time

Case Processing Summary

	Time	Cases					
		Valid		Missing		Total	
		N	Percent	N	Percent	N	Percent
MME	3 week	56	100.0%	0	0.0%	56	100.0%
	6 months	35	100.0%	0	0.0%	35	100.0%

Bootstrap Specifications

Sampling Method	Simple
Number of Samples	1000
Confidence Interval Level	95.0%
Confidence Interval Type	Percentile

Time

Descriptives

	Time			Statistic	Std. Error	Bootstrap[a]		
						Bias	Std. Error	95% Confidence Interval Lower
MME	3 weeks	Mean		25.6714	3.95612	.0698	4.1136	18.1265
		95% Confidence Interval for Mean	Lower Bound	17.7432				
			Upper Bound	33.5997				
		5% Trimmed Mean		21.7381		.5609	3.3188	16.3958
		Median		21.2500		1.0038	5.5147	15.0000
		Variance		876.448		-7.258	444.307	265.981
		Std. Deviation		29.60486		-1.07412	7.43248	16.30892
		Minimum		.00				
		Maximum		180.00				
		Range		180.00				

		Interquartile Range		32.55		-1.28	5.72	20.00
		Skewness		2.926	.319	-.703	1.023	.268
		Kurtosis		12.948	.628	-4.806	6.139	-1.109
	6 months	Mean		5.8114	1.27186	.0518	1.2872	3.4727
		95% Confidence Interval for Mean	Lower Bound	3.2267				
			Upper Bound	8.3962				
		5% Trimmed Mean		4.9690		.1640	1.2848	2.9375
		Median		2.3000		.5896	3.2023	.0000
		Variance		56.617		-1.389	18.395	23.913
		Std. Deviation		7.52442		-.19849	1.24897	4.89005
		Minimum		.00				
		Maximum		30.00				
		Range		30.00				
		Interquartile Range		10.00		-.05	1.17	7.50
		Skewness		1.448	.398	-.155	.431	.412
		Kurtosis		2.087	.778	-.558	1.750	-1.369

Descriptives

	Time			Bootstrap 95% Confidence Interval Upper
MME	3 weeks	Mean		34.7571
		95% Confidence Interval for Mean	Lower Bound	
			Upper Bound	
		5% Trimmed Mean		29.8808
		Median		30.0000
		Variance		1875.071
		Std. Deviation		43.30209
		Minimum		
		Maximum		
		Range		
		Interquartile Range		43.75
		Skewness		3.721
		Kurtosis		20.675
	6 months	Mean		8.5233
		95% Confidence Interval for Mean	Lower Bound	
			Upper Bound	
		5% Trimmed Mean		7.8187
		Median		10.0000

	Variance	94.179
	Std. Deviation	9.70461
	Minimum	
	Maximum	
	Range	
	Interquartile Range	12.20
	Skewness	2.157
	Kurtosis	5.498

a. Unless otherwise noted, bootstrap results are based on 1000 bootstrap samples

Tests of Normality

		Kolmogorov-Smirnov[a]			Shapiro-Wilk		
	Time	Statistic	df	Sig.	Statistic	df	Sig.
MME	3 weeks	.193	56	<.001	.723	56	<.001
	6 months	.266	35	<.001	.770	35	<.001

a. Lilliefors Significance Correction

Test of Homogeneity of Variance

		Levene Statistic	df1	df2	Sig.
MME	Based on Mean	11.929	1	89	<.001
	Based on Median	11.279	1	89	.001
	Based on Median and with adjusted df	11.279	1	59.493	.001
	Based on trimmed mean	11.165	1	89	.001

Kruskal-Wallis Test

Ranks

	Time	N	Mean Rank
MME	3 weeks	56	55.02
	6 months	35	31.57
	Total	91	

Test Statistics[a,b]

	MME

Kruskal-Wallis H	17.759
df	1
Asymp. Sig.	<.001

a. Kruskal Wallis Test
b. Grouping Variable: Time

Mann-Whitney Test

Ranks

	Time	N	Mean Rank	Sum of Ranks
MME	3 weeks	56	55.02	3081.00
	6 months	35	31.57	1105.00
	Total	91		

Test Statistics[a]

	MME
Mann-Whitney U	475.000
Wilcoxon W	1105.000
Z	-4.214
Asymp. Sig. (2-tailed)	<.001

a. Grouping Variable: Time
