# Supplementary material for: Assessing pain management in total joint arthroplasty using the Detroit interventional pain assessment scale—A prospective cohort study
Source: Arthroplasty. 2024 Nov 1;6:55. doi: 10.1186/s42836-024-00276-w (PMC11529018; doi:10.1186/s42836-024-00276-w)
Supplement: Supplementary file 6 — Supplementary Material 6. [file 42836_2024_276_MOESM6_ESM.pdf]

TKA difference in percentage of patients on narcotics

Time

Case Processing Summary

		Cases					
		Valid		Missing		Total	
	Time	N	Percent	N	Percent	N	Percent
Percentage_Patients	3 weeks	56	100.0%	0	0.0%	56	100.0%
	6 months	36	100.0%	0	0.0%	36	100.0%

Bootstrap Specifications

Sampling Method	Simple
Number of Samples	1000
Confidence Interval Level	95.0%
Confidence Interval Type	Percentile

Descriptives

						Bootstrap[a]	
	Time			Statistic	Std. Error	Bias	Std. Error
Percentage_Patients	3 weeks	Mean		75.0000	5.83874	-.0466	5.5928
		95% Confidence Interval for Mean	Lower Bound	63.2989			
			Upper Bound	86.7011			
		5% Trimmed Mean		77.7778		-.0518	6.2142
		Median		100.0000		.0000	.0000
		Variance		1909.091		-29.217	283.179
		Std. Deviation		43.69314		-.46626	3.36485
		Minimum		.00			
		Maximum		100.00			
		Range		100.00			
		Interquartile Range		75.00		-21.58	47.83
		Skewness		-1.187	.319	-.040	.379

		Kurtosis		-.615	.628	.250	1.079
	6 months	Mean		52.7778	8.43849	-.3619	8.5881
		95% Confidence Interval for Mean	Lower Bound	35.6467			
			Upper Bound	69.9088			
		5% Trimmed Mean		53.0864		-.4021	9.5423
		Median		100.0000		-37.5000	46.7540
		Variance		2563.492		-72.550	115.991
		Std. Deviation		50.63094		-.73630	1.21187
		Minimum		.00			
		Maximum		100.00			
		Range		100.00			
		Interquartile Range		100.00		-.45	5.90
		Skewness		-.116	.393	.010	.380
		Kurtosis		-2.107	.768	.148	.274

Descriptives

	Time			Bootstrap 95% Confidence Interval	
				Lower	Upper
Percentage_Patients	3 weeks	Mean		63.7940	85.9585
		95% Confidence Interval for Mean	Lower Bound		
			Upper Bound		
		5% Trimmed Mean		65.3267	89.9539
		Median		100.0000	100.0000
		Variance		1229.681	2350.272
		Std. Deviation		35.06678	48.47961
		Minimum			
		Maximum			
		Range			
		Interquartile Range		.00	100.00
		Skewness		-2.127	-.589
		Kurtosis		-1.713	2.614

	6 months	Mean		35.8974	69.2308
		95% Confidence Interval for Mean	Lower Bound		
			Upper Bound		
		5% Trimmed Mean		34.3305	71.3675
		Median		.0000	100.0000
		Variance		2154.318	2586.166
		Std. Deviation		46.41462	50.85437
		Minimum			
		Maximum			
		Range			
		Interquartile Range		100.00	100.00
		Skewness		-.867	.621
		Kurtosis		-2.148	-1.242

a. Unless otherwise noted, bootstrap results are based on 1000 bootstrap samples

Tests of Normality

		Kolmogorov-Smirnov[a]			Shapiro-Wilk		
	Time	Statistic	df	Sig.	Statistic	df	Sig.
Percentage_Patients	3 weeks	.466	56	<.001	.539	56	<.001
	6 months	.352	36	<.001	.636	36	<.001

a. Lilliefors Significance Correction

Test of Homogeneity of Variance

		Levene Statistic	df1	df2	Sig.
Percentage_Patients	Based on Mean	11.332	1	90	.001
	Based on Median	5.002	1	90	.028
	Based on Median and with adjusted df	5.002	1	88.085	.028
	Based on trimmed mean	11.332	1	90	.001

Kruskal-Wallis Test

Ranks

	Time	N	Mean Rank
Percentage_Patients	3 weeks	56	50.50
	6 months	36	40.28
	Total	92	

Test Statistics[a,b]

	Percentage_Patients
Kruskal-Wallis H	4.791
df	1
Asymp. Sig.	.029

a. Kruskal Wallis Test

b. Grouping Variable: Time

Mann-Whitney Test

Ranks

	Time	N	Mean Rank	Sum of Ranks
Percentage_Patients	3 weeks	56	50.50	2828.00
	6 months	36	40.28	1450.00
	Total	92		

Test Statistics[a]

	Percentage_Patients
Mann-Whitney U	784.000
Wilcoxon W	1450.000
Z	-2.189
Asymp. Sig. (2-tailed)	.029

a. Grouping Variable: Time
