# Supplementary material for: Assessing pain management in total joint arthroplasty using the Detroit interventional pain assessment scale—A prospective cohort study
Source: Arthroplasty. 2024 Nov 1;6:55. doi: 10.1186/s42836-024-00276-w (PMC11529018; doi:10.1186/s42836-024-00276-w)
Supplement: Supplementary file 7 — Supplementary Material 7. [file 42836_2024_276_MOESM7_ESM.pdf]

THA vs TKA percent of patients on narcotics

## THA_TKA

### Case Processing Summary

| | THA_TKA | Cases | | | | | |
|---|---|---|---|---|---|---|---|
| | | Valid | | Missing | | Total | |
| | | N | Percent | N | Percent | N | Percent |
| Percentage_Patients | THA | 13 | 100.0% | 0 | 0.0% | 13 | 100.0% |
| | TKA | 36 | 100.0% | 0 | 0.0% | 36 | 100.0% |

### Bootstrap Specifications

| | |
|---|---|
| Sampling Method | Simple |
| Number of Samples | 1000 |
| Confidence Interval Level | 95.0% |
| Confidence Interval Type | Percentile |

## THA_TKA

### Descriptives

| | THA_TKA | | | Statistic | Std. Error | Bootstrap[a] Bias | Bootstrap[a] Std. Error |
|---|---|---|---|---|---|---|---|
| Percentage_Patients | THA | Mean | | 15.3846 | 10.41543 | .3229 | 9.9639 |
| | | 95% Confidence Interval for Mean | Lower Bound | -7.3087 | | | |
| | | | Upper Bound | 38.0779 | | | |
| | | 5% Trimmed Mean | | 11.5385 | | .9484 | 10.2421 |
| | | Median | | .0000 | | .2000 | 3.8698 |
| | | Variance | | 1410.256 | | -75.706 | 707.685 |
| | | Std. Deviation | | 37.55338 | | -3.67335 | 13.67045 |
| | | Minimum | | .00 | | | |
| | | Maximum | | 100.00 | | | |
| | | Range | | 100.00 | | | |
| | | Interquartile Range | | .00 | | 22.70 | 37.73 |

| | | | | | | | |
|---|---|---|---|---|---|---|---|
| | | Skewness | | 2.179 | .616 | .059[b] | .996[b] |
| | | Kurtosis | | 3.223 | 1.191 | 1.507[b] | 5.631[b] |
| | TKA | Mean | | 52.7778 | 8.43849 | .1020 | 8.6756 |
| | | 95% Confidence Interval for Mean | Lower Bound | 35.6467 | | | |
| | | | Upper Bound | 69.9088 | | | |
| | | 5% Trimmed Mean | | 53.0864 | | .1134 | 9.6395 |
| | | Median | | 100.0000 | | -38.6000 | 47.2999 |
| | | Variance | | 2563.492 | | -77.644 | 122.383 |
| | | Std. Deviation | | 50.63094 | | -.78897 | 1.27582 |
| | | Minimum | | .00 | | | |
| | | Maximum | | 100.00 | | | |
| | | Range | | 100.00 | | | |
| | | Interquartile Range | | 100.00 | | -.53 | 6.05 |
| | | Skewness | | -.116 | .393 | -.011 | .384 |
| | | Kurtosis | | -2.107 | .768 | .159 | .282 |

## Descriptives

| | | | | Bootstrap 95% Confidence Interval | |
|---|---|---|---|---|---|
| | THA_TKA | | | Lower | Upper |
| Percentage_Patients | THA | Mean | | .0000 | 37.4711 |
| | | 95% Confidence Interval for Mean | Lower Bound | | |
| | | | Upper Bound | | |
| | | 5% Trimmed Mean | | .0000 | 36.0790 |
| | | Median | | .0000 | .0000 |
| | | Variance | | .000 | 2564.103 |
| | | Std. Deviation | | .00000 | 50.63697 |
| | | Minimum | | | |
| | | Maximum | | | |
| | | Range | | | |
| | | Interquartile Range | | .00 | 100.00 |
| | | Skewness | | .599[b] | 4.000[b] |
| | | Kurtosis | | -2.080[b] | 16.000[b] |

| | | | | | |
|---|---|---|---|---|---|
| | TKA | Mean | | 36.5854 | 70.5882 |
| | | 95% Confidence Interval for Mean | Lower Bound | | |
| | | | Upper Bound | | |
| | | 5% Trimmed Mean | | 35.0949 | 72.8758 |
| | | Median | | .0000 | 100.0000 |
| | | Variance | | 2087.346 | 2575.758 |
| | | Std. Deviation | | 45.68746 | 50.75192 |
| | | Minimum | | | |
| | | Maximum | | | |
| | | Range | | | |
| | | Interquartile Range | | 100.00 | 100.00 |
| | | Skewness | | -.946 | .583 |
| | | Kurtosis | | -2.129 | -1.043 |

a. Unless otherwise noted, bootstrap results are based on 1000 bootstrap samples
b. Based on 894 samples

**Tests of Normality**

| | | Kolmogorov-Smirnov[a] | | | Shapiro-Wilk | | |
|---|---|---|---|---|---|---|---|
| | THA_TKA | Statistic | df | Sig. | Statistic | df | Sig. |
| Percentage_Patients | THA | .505 | 13 | <.001 | .446 | 13 | <.001 |
| | TKA | .352 | 36 | <.001 | .636 | 36 | <.001 |

a. Lilliefors Significance Correction

**Test of Homogeneity of Variance**

| | | Levene Statistic | df1 | df2 | Sig. |
|---|---|---|---|---|---|
| Percentage_Patients | Based on Mean | 30.340 | 1 | 47 | <.001 |
| | Based on Median | 4.267 | 1 | 47 | .044 |
| | Based on Median and with adjusted df | 4.267 | 1 | 44.800 | .045 |
| | Based on trimmed mean | 30.340 | 1 | 47 | <.001 |

## Kruskal-Wallis Test

**Ranks**

| | THA_TKA | N | Mean Rank |
|---|---|---|---|
| Percentage_Patients | THA | 13 | 18.27 |
| | TKA | 36 | 27.43 |
| | Total | 49 | |

**Test Statistics[a,b]**

| | Percentage_Patients |
|---|---|
| Kruskal-Wallis H | 5.342 |
| df | 1 |
| Asymp. Sig. | .021 |

a. Kruskal Wallis Test

b. Grouping Variable: THA_TKA

## Mann-Whitney Test

**Ranks**

| | THA_TKA | N | Mean Rank | Sum of Ranks |
|---|---|---|---|---|
| Percentage_Patients | THA | 13 | 18.27 | 237.50 |
| | TKA | 36 | 27.43 | 987.50 |
| | Total | 49 | | |

**Test Statistics[a]**

| | Percentage_Patients |
|---|---|
| Mann-Whitney U | 146.500 |
| Wilcoxon W | 237.500 |
| Z | -2.311 |
| Asymp. Sig. (2-tailed) | .021 |

a. Grouping Variable: THA_TKA
