# Supplementary material for: Assessing pain management in total joint arthroplasty using the Detroit interventional pain assessment scale—A prospective cohort study
Source: Arthroplasty. 2024 Nov 1;6:55. doi: 10.1186/s42836-024-00276-w (PMC11529018; doi:10.1186/s42836-024-00276-w)
Supplement: Supplementary file 8 — Supplementary Material 8. [file 42836_2024_276_MOESM8_ESM.pdf]

Previously on Opioids vs Opioid Naïve

Case Processing Summary

	Patients	Cases					
		Valid		Missing		Total	
		N	Percent	N	Percent	N	Percent
MMEs	Previous	74	98.7%	1	1.3%	75	100.0%
	Naive	125	100.0%	0	0.0%	125	100.0%

Bootstrap Specifications

Sampling Method	Simple
Number of Samples	1000
Confidence Interval Level	95.0%
Confidence Interval Type	Percentile

Descriptives

	Patients			Statistic	Std. Error	Bootstrap[a]		
						Bias	Std. Error	95% Confidence Interval Lower
MMEs	Previous	Mean		22.8064	3.53657	-.0621	3.4951	16.2970
		95% Confidence Interval for Mean	Lower Bound	15.7580				
			Upper Bound	29.8547				
		5% Trimmed Mean		18.3134		.2682	2.8797	13.5381
		Median		15.0000		-1.1718	3.1759	10.0000
		Variance		925.542		-21.008	369.664	288.587
		Std. Deviation		30.42272		-.99301	6.20200	16.98785
		Minimum		.00				
		Maximum		180.00				
		Range		180.00				
		Interquartile Range		25.00		-.39	4.59	15.27
		Skewness		2.981	.279	-.298	.645	1.415
		Kurtosis		11.270	.552	-1.932	4.829	1.909
	Naive	Mean		13.4296	2.11104	.0261	2.0853	9.7604

	95% Confidence Interval for Mean	Lower Bound	9.2513				
		Upper Bound	17.6079				
	5% Trimmed Mean		10.2884		.0746	1.5580	7.4535
	Median		.0000		1.1458	2.4432	.0000
	Variance		557.063		-5.793	235.269	220.185
	Std. Deviation		23.60219		-.64052	4.90475	14.83865
	Minimum		.00				
	Maximum		180.00				
	Range		180.00				
	Interquartile Range		26.25		-1.35	5.70	15.00
	Skewness		3.849	.217	-.616	1.058	1.007
	Kurtosis		21.973	.430	-5.783	9.298	-.237

Descriptives

	Patients			Bootstrap 95% Confidence Interval Upper
MMEs	Previous	Mean		30.2445
		95% Confidence Interval for Mean	Lower Bound	
			Upper Bound	
		5% Trimmed Mean		24.8627
		Median		20.0000
		Variance		1729.536
		Std. Deviation		41.58769
		Minimum		
		Maximum		
		Range		
		Interquartile Range		36.23
		Skewness		3.963
		Kurtosis		20.390
	Naive	Mean		17.7972
		95% Confidence Interval for Mean	Lower Bound	
			Upper Bound	
		5% Trimmed Mean		13.5772
		Median		7.5000
		Variance		1064.523
		Std. Deviation		32.62699
		Minimum		

	Maximum	
	Range	
	Interquartile Range	30.00
	Skewness	4.776
	Kurtosis	34.563

a. Unless otherwise noted, bootstrap results are based on 1000 bootstrap samples

Tests of Normality

		Kolmogorov-Smirnov[a]			Shapiro-Wilk		
	Patients	Statistic	df	Sig.	Statistic	df	Sig.
MMEs	Previous	.231	74	<.001	.679	74	<.001
	Naive	.285	125	<.001	.589	125	<.001

a. Lilliefors Significance Correction

Test of Homogeneity of Variance

		Levene Statistic	df1	df2	Sig.
MMEs	Based on Mean	1.464	1	197	.228
	Based on Median	1.445	1	197	.231
	Based on Median and with adjusted df	1.445	1	195.426	.231
	Based on trimmed mean	1.227	1	197	.269

Kruskal-Wallis Test

Ranks

	Patients	N	Mean Rank
MMEs	Previous	74	118.06
	Naive	125	89.31
	Total	199	

Test Statistics[a,b]

	MMEs
Kruskal-Wallis H	12.533
df	1
Asymp. Sig.	<.001

a. Kruskal Wallis Test
b. Grouping Variable: Patients

Mann-Whitney Test

Ranks

	Patients	N	Mean Rank	Sum of Ranks
MMEs	Previous	74	118.06	8736.50
	Naive	125	89.31	11163.50
	Total	199		

Test Statistics[a]

	MMEs
Mann-Whitney U	3288.500
Wilcoxon W	11163.500
Z	-3.540
Asymp. Sig. (2-tailed)	<.001

a. Grouping Variable: Patients
